# Perceptions and experiences of the implementation, management, use and optimisation of electronic prescribing systems in hospital settings: protocol for a systematic review of qualitative studies

**DOI:** 10.1136/bmjopen-2016-011858

**Published:** 2016-07-08

**Authors:** Albert Farre, Danai Bem, Gemma Heath, Karen Shaw, Carole Cummins

**Affiliations:** 1 Institute of Applied Health Research, University of Birmingham, Birmingham, UK; 2 Research and Development, Birmingham Children's Hospital NHS Foundation Trust, Birmingham, UK; 3 School of Life and Health Sciences, Aston University, Birmingham, UK

**Keywords:** Electronic Prescribing, Computerized Physician Order Entry, Systematic Review, QUALITATIVE RESEARCH, Thematic Synthesis

## Abstract

**Introduction:**

There is increasing evidence that electronic prescribing (ePrescribing) or computerised provider/physician order entry (CPOE) systems can improve the quality and safety of healthcare services. However, it has also become clear that their implementation is not straightforward and may create unintended or undesired consequences once in use. In this context, qualitative approaches have been particularly useful and their interpretative synthesis could make an important and timely contribution to the field. This review will aim to identify, appraise and synthesise qualitative studies on ePrescribing/CPOE in hospital settings, with or without clinical decision support.

**Methods and analysis:**

Data sources will include the following bibliographic databases: MEDLINE, MEDLINE In Process, EMBASE, PsycINFO, Social Policy and Practice via Ovid, CINAHL via EBSCO, The Cochrane Library (CDSR, DARE and CENTRAL databases), Nursing and Allied Health Sources, Applied Social Sciences Index and Abstracts via ProQuest and SCOPUS. In addition, other sources will be searched for ongoing studies (ClinicalTrials.gov) and grey literature: Healthcare Management Information Consortium, Conference Proceedings Citation Index (Web of Science) and Sociological abstracts. Studies will be independently screened for eligibility by 2 reviewers. Qualitative studies, either standalone or in the context of mixed-methods designs, reporting the perspectives of any actors involved in the implementation, management and use of ePrescribing/CPOE systems in hospital-based care settings will be included. Data extraction will be conducted by 2 reviewers using a piloted form. Quality appraisal will be based on criteria from the Critical Appraisal Skills Programme checklist and Standards for Reporting Qualitative Research. Studies will not be excluded based on quality assessment. A postsynthesis sensitivity analysis will be undertaken. Data analysis will follow the thematic synthesis method.

**Ethics and dissemination:**

The study does not require ethical approval as primary data will not be collected. The results of the study will be published in a peer-reviewed journal and presented at relevant conferences.

**Trial registration number:**

CRD42016035552.

**Strengths and limitations of this study**

- Although a number of systematic reviews have been conducted to date on electronic prescribing (ePrescribing) or computerised provider/physician order entry systems, only a few have focused on hospital settings.
- According to the scoping searches conducted by the authors, only two existing reviews included qualitative studies, of which only one focused on providers' perceptions in a hospital setting.
- To the best of the knowledge of the authors, this is the first systematic review of qualitative evidence relating to the management and use/optimisation of ePrescribing systems in hospital settings, not limited to the implementation process, and including any reported perspectives (ie, not only health professionals and also managers, commissioners, patients and relatives/carers).
- The review will not address perceptions and experiences related to non-hospital-based settings.

## Introduction

In recent years, an increasing body of evidence supports that electronic prescribing (ePrescribing) or computerised provider/physician order entry (CPOE) systems can improve medication safety and quality of healthcare services by reducing risks of medication errors^1^ ^2^ and improving organisational efficiency and health professionals' performance throughout the medication process.^2^ ^3^

However, alongside the potential benefits of ePrescribing applications for patients, professionals and healthcare systems/organisations, it has also become clear that their implementation is not straightforward and may create unintended or undesired consequences once in use,^3–6^ with potentially adverse effects on clinical workflows^7^ and collaborative work^8^ such as increased data entry tasks and redistribution of work and time for patient care^9^ or reduced interactions between nurses and doctors.^10^ Likewise, as work practices change, hospital staff have to learn new ways of working, which can also result in the development of ‘workarounds’ where technology is used in ways other than intended.^11–13^ This, together with the inherent difficulty of demonstrating the impact of health information technologies (HITs) on clinical outcomes^2^ ^14^ due to the systemic nature of changes brought about by HIT implementation, means that inductive exploratory approaches of a qualitative nature can make an important contribution to this field.^9^ ^15–17^

Qualitative approaches are particularly well suited to (1) understanding how systems are used in practice, including the identification of unintended consequences, to inform the implementation, development and optimisation of ePrescribing systems in specific contexts;^3^ ^18–21^ (2) considering the role of social and organisational factors alongside technology in the implementation process;^8^ ^12^ ^22^ and (3) making sense of complex data about quality and safety, which cannot be straightforwardly classified or quantified and therefore cannot be elicited from sources such as audit data, routine metrics and statistics or incident-reporting systems.^23–26^

On this basis, the proposed systematic review will collate, assess and analyse the current evidence relating to perceptions and experiences of those involved in the implementation, management, use and optimisation of ePrescribing systems in the hospital setting.

A number of systematic reviews have been conducted to date on ePrescribing/CPOE systems: five reviews used quantitative approaches^27–31^ and two used qualitative approaches,^32^ ^33^ of which only one focused on user perceptions of ePrescribing system implementation in a hospital setting.^33^ However, this review was restricted to health professionals' perceptions of implementation (ie, barriers and facilitators to the implementation process) and therefore did not include (1) any experiences relating to the use of ePrescribing systems beyond the ‘implementation stage’ and (2) any experiences from other actors involved in and/or affected by ePrescribing such as service managers, commissioners, patients and relatives/carers. In addition, there were some methodological limitations to this review such as the adoption of an aggregative (rather than interpretative) approach to data synthesis, language restrictions and exclusion of poor quality studies. Thus, given the very limited number of primary studies previously identified (n=5), a systematic review of qualitative studies with a broader focus and an interpretative approach is needed. It is anticipated that this will move the field forward by deepening our conceptual understandings of the implementation, management, use and optimisation of ePrescribing from the perspectives of those involved.

### Aims and objectives

The aim of this review is to explore the perceptions and experiences of the implementation, management, use and optimisation of ePrescribing/CPOE systems with or without clinical decision support (CDS) in the hospital setting.

#### Objectives

- To identify, appraise and synthesise primary qualitative studies on ePrescribing/CPOE systems, with or without CDS, in hospital settings.
- To explore/interpret the contribution of such findings to the understanding of the perceptions and experiences of those involved in the implementation, management, use and optimisation of ePrescribing/CPOE systems in hospital settings.

## Methods and analysis

### Design

This systematic review will follow the reporting guidelines formulated in the ‘Enhancing transparency in reporting the synthesis of qualitative research’ (ENTREQ) statement.^34^

#### Search strategy

The following sources will be searched for primary studies:

- Bibliographic databases: MEDLINE, MEDLINE In Process, EMBASE, PsycINFO, Social Policy and Practice via Ovid, CINAHL via EBSCO, The Cochrane Library (CDSR, DARE and CENTRAL databases), Nursing and Allied Health Sources, Applied Social Sciences Index and Abstracts (ASSIA) via ProQuest and SCOPUS.
- Sciences and Social Sciences Citation Index (Web of Science) for citation searching.
- ClinicalTrials.gov for ongoing studies.
- Checking of citation lists of included studies and relevant reviews.
- Grey literature sources: Healthcare Management Information Consortium, Conference proceedings citation index (Web of Science) and Sociological abstracts.
- Selected specialist journals in the field of health informatics and qualitative research will be hand searched for additional relevant studies.

A comprehensive search strategy will be conducted with the use of search filters^35^ ^36^ or Medical Subject Headings terms relating to qualitative and mixed-methods research where appropriate in order to identify relevant qualitative studies. A combination of text words and index terms will be used for each of the two main sets of concepts relating to the perspective (including terms such as perceptions, attitudes, views and experiences) and intervention (including terms such as ePrescribing, CPOE and CDS) including variations and permutations of search terms tested and used in previous systematic reviews with a similar scope.^2^ ^33^ ^37–39^ There will be no restriction on date or language of publication. A sample search strategy for MEDLINE is provided (see online supplementary appendix 1) and this strategy has been adapted for use in each bibliographic database.

10.1136/bmjopen-2016-011858.supp1 supplementary appendix

#### Selection criteria

The literature search results will be imported into EndNote X7.4 (Thomson Reuters, New York) in order to remove duplicate records and complete the study selection process. Two reviewers will independently screen articles for eligibility using predetermined inclusion/exclusion criteria (table 1). Any discrepancies between reviewers will be resolved by discussion or with the involvement of a third reviewer where consensus was not reached. Translation of non-English language articles will be undertaken if necessary. The selection process will be illustrated using a Preferred Reporting Items for Systematic Reviews and Meta-Analyses flow diagram.^40^

**Table 1:** Inclusion and exclusion criteria for study selection

Include	Exclude
Study design	
Qualitative studies, standalone Qualitative studies in the context of mixed-methods or other designs Systematic reviews of qualitative studies (at least one database searched)	Quantitative studies Clearly commentary/letter only with no new data from primary studies Clearly narrative review (no mention of any database searched)
Publication type	
Full article Letter Conference abstracts Study report	Letter with a commentary on other studies only
Setting	
Any hospital-based care setting Include any mixed-care setting at this stage	Primary care settings Clinics not hospital-based Secondary/tertiary care not hospital-based Community care not hospital-based Residential care setting
Perspective—population	
All individuals involved in electronic prescribing systems' implementation, use and management such as: service providers (including doctors, nurses, pharmacists and allied health professionals), service users (including patients and family members or relatives of patients), personnel involved in the management and/or implementation, policymakers or commissioners responsible for the introduction of such systems	None
Intervention	
Any electronic system, or subsystem, involved in the prescription and/or administration phase of the medication process	Any electronic system not involved in the prescription and/or administration phases of the medication process (eg, robots that pick drugs for dispensing and systems for stock control are excluded)
Evaluation—outcomes	
No restriction by outcome	

#### Setting

Any hospital-based care setting will be included. Other settings such as primary care, non-hospital-based clinics, non-hospital-based secondary/tertiary care, non-hospital-based community care and residential care settings will be excluded. In cases where a study includes non-hospital-based and hospital-based settings, a decision regarding study inclusion will be made on the basis of the reported data that belong to the hospital side of the study being identifiable and extractable.

#### Perspective

This review will not focus on a particular perspective, and therefore, studies of any types of participants will be included. Participants/perspectives included in the review may comprise, but are not limited to, the following: service providers (including doctors, nurses, pharmacists and any allied health professionals who are involved in the use of ePrescribing systems), service users (including patients and relatives/carers of patients), personnel involved in the management and/or implementation of ePrescribing systems and policymakers or commissioners responsible for the introduction of ePrescribing systems.

#### Intervention

Any electronic system, or subsystem, involved in the prescription and/or administration of medication will be included. This comprises studies of CDS systems within or alongside ePrescribing/CPOE and studies of electronic health records systems that include ePrescribing/CPOE. In the latter case, studies will only be included if the ePrescribing/CPOE-related data are identifiable and extractable. Electronic systems only involved in other phases of the medication process (documenting/transcribing, dispensing or monitoring) will be excluded.

#### Comparison

No restrictions applied.

#### Evaluation

Any qualitative studies focusing on the perceptions and experiences of those involved in the implementation, management, use and optimisation of ePrescribing systems in a hospital setting. The main outcome of this review will be to identify key issues and areas of importance from the users' perspectives to inform the quality and safety of healthcare services delivery.

#### Quality appraisal

Quality appraisal of included studies will be conducted using a tool developed specifically for the review based on items from the Critical Appraisal Skills Programme Qualitative Research Checklist,^41^ and the Standards for Reporting Qualitative Research.^42^ The methodological quality of each study will be appraised by two reviewers independently and the outcome and reasons for their assessments recorded. Disagreements will be resolved by discussion or referral to a third reviewer.

Given the inherent difficulty of appraising all aspects of quality of qualitative research,^43^ studies will not be excluded based on quality or adequacy of the reporting. Instead, a postsynthesis sensitivity analysis^44^ will be undertaken to explore whether any particular finding or group of findings is dependent, either exclusively or disproportionately, on one or more studies classified as ‘low quality’ or ‘inadequately reported’ by the review team. The quality appraisal and sensitivity analysis will enable an accurate judgement of the quality of the evidence included in the review, which will be discussed and considered in the reporting of the findings. In addition, it will indicate any issues shared by studies that should be considered when designing any new qualitative studies.

#### Data extraction

Data extraction will be conducted by two reviewers using a standardised piloted data extraction form. Discrepancies will be resolved through discussion or referral to a third reviewer. The following (but not limited to) information will be extracted from all included studies:

- Purpose of the study and specific objectives or research questions;
- Study characteristics, study location (eg, country, setting);
- Population (eg, number, age, gender, recruitment/sampling strategies);
- Qualitative approach and research paradigm (eg, grounded theory, ethnography, phenomenology);
- Data collection methods;
- Data analysis methods;
- Results.

We will understand the findings to be all of the text labelled as ‘results’ or ‘findings’ in the selected papers. Included data will therefore include verbatim data extracts from participants as well as authors' descriptions, summaries and interpretation of primary data. These will also be used to contextualise and facilitate the interpretation of primary findings included in the review.

### Data analysis

The results sections of the included studies will be entered, verbatim, into QSR NVivo 10 software and will be treated as qualitative data.

We will then undertake a thematic synthesis,^45^ which we consider particularly suitable for this work, where included studies are likely to bring in a wide range of intervention features (systems' scope and functionalities), settings (including different countries) and perspectives that could hinder the potential for conceptual innovation. A key strength of using thematic analysis is the flexibility that this method offers in this respect, that is, it is possible to synthesise with or without conceptual innovation using thematic synthesis. While the review team will be aiming for an analytical (rather than descriptive) synthesis output, if establishing analytical themes were to be deemed problematic by the review team, it would still be possible to produce a satisfactory synthesis using this method. Also, thematic synthesis has been regarded as a suitable and relevant method for synthesising qualitative research in the context of health technology assessment.^46^

Following Thomas and Harden,^45^ this will involve three overlapped and inter-related stages: (1) line-by-line coding of the findings of the primary studies, (2) categorisation of codes into descriptive themes and (3) development of analytical themes to describe and/or explain all of the previous descriptive themes. As we will adopt an inductive approach to data analysis, the main analytical concern of the review has not been established beforehand. During the initial descriptive stage of the data analysis process, we expect to identify the main issues reported by primary studies alongside the range of aspects that frame them, including perspectives/types of participants, implementation stages, system functionality and level of care, that might be deemed relevant once the reviewers achieve familiarity with the extracted primary data. These will then be critically discussed and put in relation to the main emerging descriptive themes to then consider the focus of the analytical stage of the thematic synthesis.

To ensure the robustness of the findings, various verification procedures will be undertaken including:

- Multiple coding: data will be codified independently by two reviewers.
- Reviewer triangulation: three reviewers will compare their interpretations of the two codebooks.
- Data sessions: these will be held at various stages and will involve all members of the review team to (1) discuss and agree on the coding framework; (2) establish and refine descriptive themes; (3) discuss and agree analytical themes and interpretative framework and (4) discuss any emerging issues from the sensitivity analysis and establish final results.

## Discussion

Those involved in and/or affected by technologies aimed at supporting the medication prescription and administration process in hospital settings will include not only health professionals and also other actors such as patients and relatives/carers, managers, commissioners or HIT professionals.

A better understanding of the range of actors and their perspectives of implementing and using ePrescribing technologies in hospital settings could help to develop new measures of impact for future research and quantitative impact evaluations, and offer new insights into the implementation process of such technologies by bringing together perspectives from strategic, social, organisational and technical backgrounds.^8^ ^22^

Also, this work might open up new angles from which to consider key findings from previous similar reviews in this area, including the introduction of profound workflow changes (such as staff communication patterns or dependence on technology) and new safety hazards (such as system workarounds or alert fatigue),^32^ as well as implementation barriers (such as poor access to computers or unsupportive management teams) and facilitators (such as teamwork and user involvement or adequate staff training).^33^

In addition, the analysis of existing qualitative evidence on the implementation and regular use of ePrescribing in hospital settings from the perspective of those involved in and/or affected by such technologies could shed new light on the issue of addressing complex data about healthcare quality and safety that cannot be straightforwardly classified, quantified or elicited from sources such as audit data, routine metrics and statistics or incident-reporting systems.^23^ ^26^ ^47^

Finally, the findings of this review will inform an ongoing longitudinal study exploring the effects of implementing an ePrescribing system on care provision and hospital work in paediatric hospital ward settings.^48^
