# Supplementary material for: Perceptions and experiences of the implementation, management, use and optimisation of electronic prescribing systems in hospital settings: protocol for a systematic review of qualitative studies
Source: BMJ Open. 2016 Jul 8;6(7):e011858. doi: 10.1136/bmjopen-2016-011858 (PMC4947719; doi:10.1136/bmjopen-2016-011858)
Supplement: supplementary appendix [file bmjopen-2016-011858supp_appendix.pdf]

Appendix 1

Database: Ovid MEDLINE(R) <1946 to Present>

Search strategy:

1 Electronic Prescribing/

2 ("electronic prescribing" or e-prescribing or eprescribing).ti,ab.

3 Medical Order Entry Systems/ or Medication Systems, Hospital/ or Pharmacy service, Hospital/

4 ((medica$ or electronic or prescri$) adj3 system$1).ti,ab.

5 (computeri?ed adj2 ("order entry" or "order management")).ti,ab.

6 (CPOE or EP or EPMA or HEPMA or CDS or CDSS or eRx).ti,ab.

7 clinical decision support.mp. or Decision Support Systems, Clinical/

8 1 or 2 or 3 or 4 or 5 or 6 or 7

9 (perception$1 or experience$1 or perspective$1 or insight$1 or view$1 or opinion$1 or thought$1 or expectation$1 or belie$ or anticipation$1 or attitude$1 or prospect$1 or presumption$1 or observation$1 or lesson$1 or interaction$1).ti,ab.

10 8 and 9

11 limit 10 to "qualitative (maximizes specificity)"

12 qualitative research.mp. or exp Qualitative Research/

13 focus group$1.mp. or exp Focus Groups/

14 nursing methodology research.mp. or exp Nursing Methodology Research/

15 (qualitative or "grounded theory" or ethnogra$ or ethnolog$ or phenomenogra$ or phenomenolog$ or hermeneutic$ or "focus group$1" or "field study" or fieldwork or "field work" or narrat$ or "lived experience$1" or "life experience$1" or "key informant$1" or "mixed method$" or "multi-method$").ti,ab.

16 (("semi-structured" or semistructured or unstructured or informal or "in-depth" or indepth or "face-to-face" or structured or guide$) adj3 (interview$1 or discussion$1 or questionnaire$1)).ti,ab. (

17 12 or 13 or 14 or 15 or 16

18 10 and 17

19 11 or 18
